# The First Complete Chloroplast Genome of *Cordia monoica*: Structure and Comparative Analysis

**DOI:** 10.3390/genes14050976

**Published:** 2023-04-26

**Authors:** Rana M. Alshegaihi, Hassan Mansour, Shouaa A. Alrobaish, Najla A. Al Shaye, Diaa Abd El-Moneim

**Affiliations:** 1 Department of Biology, College of Science, University of Jeddah, Jeddah 21493, Saudi Arabia; rmalshegaihi@uj.edu.sa; 2 Department of Biological Sciences, Faculty of Science & Arts, King Abdulaziz University, Rabigh 21911, Saudi Arabia; hmansor@kau.edu.sa; 3 Department of Botany and Microbiology, Faculty of Science, Suez Canal University, Ismailia 41522, Egypt; 4 Department of Biology, College of Science, Qassim University, Buraydah 52377, Saudi Arabia; sh.alrobish@qu.edu.sa; 5 Department of Biology, College of Science, Princess Nourah bint Abdulrahman University, P.O. Box 84428, Riyadh 11671, Saudi Arabia; 6 Department of Plant Production, (Genetic Branch), Faculty of Environmental Agricultural Sciences, Arish University, El-Arish 45511, Egypt; dabdelmoniem@aru.edu.eg

**Keywords:** *Cordia monoica*, chloroplast genome, phylogenetic, divergence, comparative analysis

## Abstract

*Cordia monoica* is a member of the Boraginaceae family. This plant is widely distributed in tropical regions and has a great deal of medical value as well as economic importance. In the current study, the complete chloroplast (cp) genome of *C. monoica* was sequenced, assembled, annotated, and reported. This circular chloroplast genome had a size of 148,711 bp, with a quadripartite structure alternating between a pair of repeated inverted regions (26,897–26,901 bp) and a single copy region (77,893 bp). Among the 134 genes encoded by the cp genome, there were 89 protein-coding genes, 37 transfer RNA (tRNA) genes, and 8 ribosomal RNA (rRNA) genes. A total of 1387 tandem repeats were detected, with the hexanucleotides class making up 28 percent of the repeats. *Cordia monoica* has 26,303 codons in its protein-coding regions, and leucine amino acid was the most frequently encoded amino acid in contrast to cysteine. In addition, 12 of the 89 protein-coding genes were found to be under positive selection. The phyloplastomic taxonomical clustering of the Boraginaceae species provides further evidence that chloroplast genome data are reliable not only at family level but also in deciphering the phylogeny at genus level (e.g., *Cordia*).

## 1. Introduction

In green plants, chloroplasts (cp) play essential roles in photosynthesis, as well as carbon fixation, as they transform light energy into chemical energy. They are formed by photosynthetic bacteria that interact with non-photosynthetic hosts through endosymbiosis [1,2,3,4]. In addition to producing starch, amino acids, lipids, vitamins, and pigments in flowers, chloroplasts also participate in several sulfur and nitrogen metabolism pathways [5]. There are 110–130 genes encoded in the chloroplast genomes, whose size ranges from 120–180 kb, while gene order and content are highly conserved [6,7,8,9]. Most angiosperms display a quadripartite cyclic structure consisting of two identical inverted repeats (IR) separated by a large or small single-copy region (LSC and SSC, respectively) [7,9]. It has also been reported that several angiosperm lineages have undergone large-scale genome rearrangements and gene losses [10,11].

As angiosperm chloroplast genomes exhibit uniparental inheritance, stable structures, and moderate evolutionary rates, they offer sufficient genetic markers to conduct genome-wide evolutionary studies [12,13,14]. In the era of high-throughput sequencing technologies, we have been able to sequence complete genomes and analyze whole plastomes. As a result, large amounts of valuable information can be gathered, and phylogenomic analyses based on the whole plastomes can be conducted rather than specific loci [15,16,17].

Cordias are deciduous shrubs or trees belonging to the subfamily Cordioideae of the Boraginaceae family, which was previously distinguished as a separate family known as Cordiacea (Tropicos.org). Approximately 300 species are known to exist in both hemispheres, including Mexico, Central America, South America, the Arabian Peninsula, Pakistan, Sri Lanka, India, East and West Africa, Nigeria, and Ghana [18]. The tree grows up to 6 m tall and it bears white flowers and yellow fruit, with ovate leaves that can reach up to four inches long. Typically, the fruit measures between 0.5 and 1 inch long, and the flowering and fruiting process occur between October and December [19]. It has been reported that many species of *Cordia* have been used in traditional medicine for centuries to treat a range of ailments, including *C. monoica*, which showed significant anti-ulcer activity. Additionally, *C. monoica* leaves are used as a vapor bath for leprosy, its roots for vomiting, and its stem bark for chest pains [18,20,21,22,23]. Currently, there are only a few species of Boraginaceae chloroplast genome in GenBank, while *C. monoica* has never been sequenced. It remains important to further research on this family of chloroplast genomes, since significant variations are observed in the length of chloroplast genomes sequences. For example, *Pholisma arenarium* (GenBank accession: NC_039719) and *Lennoa madreporoides* (GenBank accessions: NC_039720) show 81,198 and 83,675 bp of length, respectively.

This study aimed to sequence, characterize, and compare the whole chloroplast genome sequence of *C. monoica* with other species belonging to the family Boraginaceae. By evaluating interspecific variation among genera of Boraginaceae family, it is possible to develop markers and distinguish Boraginaceae species using newly generated chloroplast genomes.

## 2. Materials and Methods

### 2.1. Sample Collection and DNA Extraction

Fresh leaves of *C. monoica* were collected from the Faifa mountains in the Jazan province of Saudi Arabia (17°15′ N 43°06′ E). Harvested fresh leaves were immediately placed in a container with silica gel and stored at 4 °C for further DNA extraction. Genomic DNA was extracted using WizPrep™ gDNA Mini Kit (Cell/Tissue, Seol, Republic of Korea), and the DNA concentration and quality were assessed using Quantus™ Fluorometer (Promega, Madison, WI, USA) and electrophoresis on a 1% agarose gel, respectively.

### 2.2. Cp-Genome Sequencing, Assembly, and Annotation

Following the instruction of the library construction kit, the purified high-quality genomic DNA was used to construct paired-end libraries by shearing the genomic DNA into short fragments of approximately 350 bp before sequencing in 150 bp paired-end mode was implemented on an Illumina HiSeq 4000 (Novogene Technologies, Beijing, China). Adapters and low-quality sequences were removed from raw reads to obtain high-quality reads. Clean filtered reads were de novo assembled using the single-contig approach [24,25]. GeSeq was used to annotate the assembled chloroplast genome [26], while Organellar Genome DRAW (OGDRAW) [27] mapped the chloroplast genome of *C. monoica.* The tRNA scan-SE 2.0 search server was used to confirm all tRNAs [28]. Geneious Prime was used to check and correct annotations and coding sequences [29].

### 2.3. Genome Analysis, Codon Usage, and Tandem Repeats Structures

SNPs and indels were detected using Geneious Prime in LSC, SSC, and IR regions. MEGA 11 software [30] was used to analyze the codon usage frequency and relative synonymous codon usage (RSCU) in *C. monoica* for all protein-coding genes. The Phobos V3.3 software was used to detect tandem repeats in CP genome sequences, implemented in Geneious Prime.

### 2.4. Sequence Divergence in Boraginaceae Family and Region Boundaries

The complete chloroplast genome of *C. monoica* was compared with other Boraginaceae species available in the GenBank database, namely, *P. arenarium*, *L. madreporoides*, *B. officinalis*, and *O. fuyunensis*, using the mVISTA program set for a shuffle-LAGAN model [31], with *C. monoica* cp genome as the reference. The chloroplast genome borders of LSC, SSC, and IRs were compared according to their annotations using IRScope online tool (https://irscope.shinyapps.io/irapp/, accessed on 12 October 2022).

### 2.5. Synonymous (dS) and Non-Synonymous (dN) Substitution Rate Analysis

To identify the genes under selection pressure, the nonsynonymous (dN), synonymous (dS), and dN/dS (ω) ratio of each protein-coding gene were used. The conditions for a positive, neutral, or purifying selection were indicated when ω  >  1, ω  =  1, and ω  <  1, respectively [32,33].

### 2.6. Phylogenetic Analyses

The phylogenetic analysis was based on the LSC region, the SSC region, and the IR region of *C. monoica* and other species of Boraginaceae downloaded from the GenBank database. Using MAFFT [34], the chloroplast genome sequences of all five species were aligned. Alignments were adjusted manually and concatenated to construct a phylogenetic tree. The phylogenetic analyses were generated using maximum likelihood (ML), computed using FastTree V2 [35], which performed under the generalized time reversible (GTR) model using the default settings; and the maximum parsimony (MP) computed on all sites using MEGA 11, adjusted to the default parameters.

## 3. Results

### 3.1. Complete Chloroplast Genome Sequence of C. monoica

**Figure 1:**
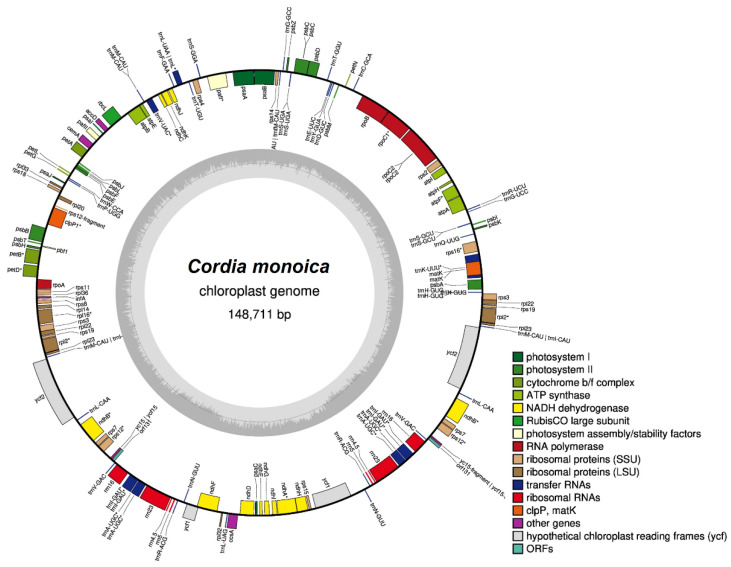
Gene map of the chloroplast genome of *C. monoica*. The genes shown on the inside and the outside of the outer circle are transcribed in clockwise and counterclockwise directions, respectively. The colored bars denote gene functional groups. The dark gray area in the inner circle indicates the GC content, while the light grey area represents the AT content. (*) label genes with introns.

The complete cp genome of *C. monoica* shows a length of 148,711 bp and a quadripartite structure typical of angiosperms. The molecule consists of a pair of inverted repeats (IRA and IRB) regions (26,897–26,901 bp). The IRA region is separated from the IRB region by a small single copy region (17,020 bp) and a large single copy (77,893 bp) region (Figure 1). A total of 134 genes are found in the cp genome, including 8 ribosomal RNA (rRNA) genes, 37 transfer RNA (tRNA) genes, and 89 protein-coding genes (PCGs). Of these, there are 22 intron-containing genes, 2 (clpP, and pafI) of which contain two introns, and 20 (13 PCGs and seven tRNAs) have one intron. The remaining 21 genes, namely, ndhB, rpl2, rpl22, rps3, rps7, rps12, rps19, ycf2, rrn4.5, rrn5, rrn16, rrn23, trnA^UGC^, trnI^CAU^, trnL^CAA^, trnR^ACG^, trnN^GUU^, trnR^ACG^, and trnV^GAC^ are duplicated in the IR regions. Notably, the *C. monoica* cp genome has the rps12 gene trans-spliced with the 3′ end duplicated in the IR regions and 5′ end in the LSC region.

It is found that the chloroplast genome contains 38.20% GC, while the LSC, SSC, and IR areas contain 36.3%, 32.1%, and 42.7%, respectively. The nucleotide frequency is 30.6% for A, 19.5% for C, 18.7% for G, and 31.3% for T. Over half of the cp genome (60.9%) is occupied by the coding region (90,615 bp) with the CDS (78,796 bp, 52.98%) regions forming the largest portion, followed by rRNA genes (9050 bp; 6.08%) and tRNA genes (2769 bp; 1.86%). The remaining 39.0% consists of intergenic regions, introns, and pseudogenes (Table 1).

### 3.2. Tandem Repeats Sequence

The *C. monoica*, *B. officinalis*, and *O. fuyunensis* chloroplast genomes were examined and show a total of 1387 tandem repeats in the noncoding regions, with a repeat unit ranging from 8 to 86 bp. Repeats are found predominantly in the LSC region (61%), while low proportions are found in the IR (31%), and SSC (8%) regions. Interestingly, most of the dinucleotide repeats belong to the AT type (67%), and the majority of other repeat classes are especially rich in A or T.

According to the repeated class, the hexanucleotide (28%) is the most abundant class of repeats followed by pentanucleotide (20%), tetranucleotide (17%), trinucleotide (14%), dinucleotide (8%), hepta-nucleotide (6%), octa-nucleotide (3%), nona-nucleotide (2%), and deca-nucleotide (1%; Table 2). The highest abundance motifs in trinucleotide repeats are AAG and AAT, AAAT motif in tetranucleotide, AAAAT in pentanucleotide, AAATAG in hexanucleotide, AAAAAAT in hepta-nucleotide, AAAAAAAT in octa-nucleotide, and AAATGTTCC in nona-nucleotide.

### 3.3. Codon Usage Bias of C. monoica

**Figure 2:**
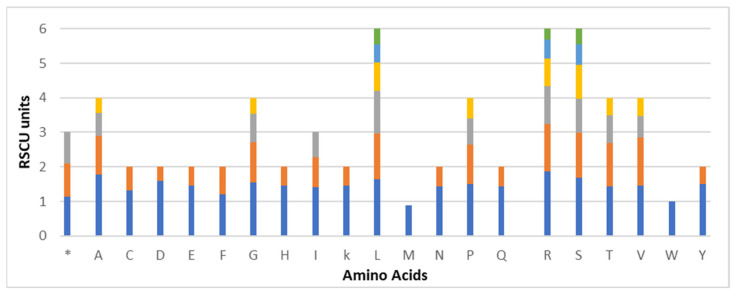
Relative synonymous codon usage (RSCU) of 20 amino acids and stop codons of the *C. monoica* chloroplast genome. * = stop codons.

Using the sequences of protein-coding genes, the frequency of codon usage for *C. monoica* cp was calculated. Using a standard set of 64 codons, 26,303 codons were used to code 20 types of amino acids. All amino acids, except methionine and tryptophan, display codon preferences. Arginine, serine, and leucine are encoded by six codons each, while the remaining amino acids are encoded by two or four codons. There are 2766 codons containing leucine (10.5%), compared with 302 codons containing cysteine (1.1%). The RSCU values of all codons are shown in Figure 2. With 29 codons with RSCU > 1, all ending in A/U except for UUG, the A/U contents are mostly observed in the third codon position. No bias in the frequency of AGU and UGG codons encoding for serine and tryptophan is observed (RSCU  =  1).

### 3.4. Comparative Analysis of Chloroplast Genome in Boraginaceae Family

**Figure 3:**
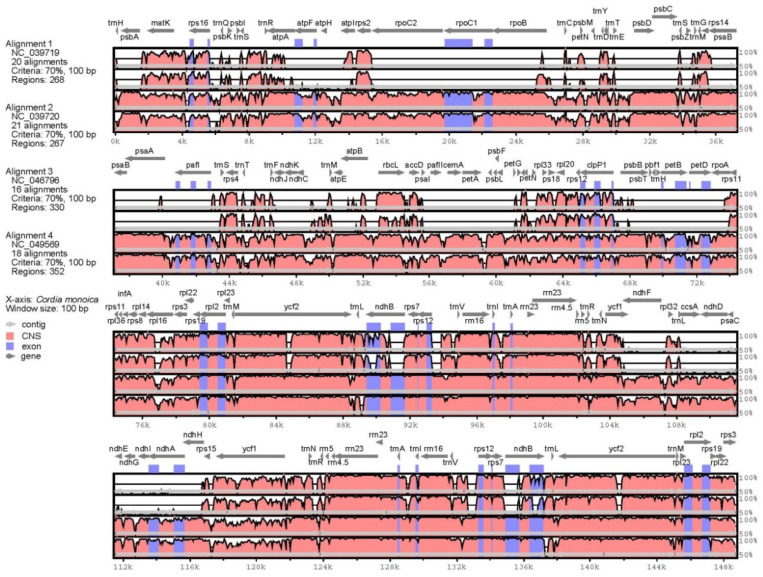
The mVista chart compares four Boraginaceae species to *C. monoica*. Genes are annotated in gray arrows; exons are highlighted in blue. Continuous regions are marked as contigs with arrows within each track. One track represents a species, 1: *P. arenarium*, 2: *L. madreporoides*, 3: *B. officinalis*, and 4: *O. fuyunensis*.

The chloroplast alignment indicates numerous changes between *C. monoica* and related species (*P. arenarium*, *L. madreporoides*, *B. officinalis*, and *O. fuyunensis*). The main variations found in the cp genomes length are, therefore, differences in the length of each region and the positioning of its boundaries (Table 3). The size cp genome ranges from 81,198 bp (*P. arenarium*) to 150,612 bp (*O. fuyunensis*). A significant difference is observed among the studied species of the family Boraginaceae. In two of the five species (*P. arenarium* and *L. madreporoides*), a severe reduction in the cp length is detected, 60% less in length compared *C. monoica*, yet the four cp regions are possibly annotated. The reduction is asymmetric among all regions; the LSC and SSC sever the major parts in contrast to the IR regions, that show high level of conservation and only 17.3% less than the IR region length in *C. monoica*. Based on the mVista, the missing regions contain coding genes, including ATP subunits, RNA polymerase genes, photosystem I, II, assembly and stability factors, and NADH dehydrogenase subunits (Figure 3).

Thus, we focused on the non-gapped regions to define the hypervariable regions of the studies’ cp genomes. The coding genes *mat*K, *rbc*L, and *rpl*16, the non-coding regions rps16 intron, and the intergenic spacers *rps*18-*rpl*22, *trn*M-*ycf*2, *rps*15-*ycf*1, *ycf*1, *trn*V-*rps*12, and *trn*M-*rpl*23 show the lowest similarity percentage among the four Boraginaceae species compared to *C. monoica*. The massive variances leads to the exclusion of two distinct species (*P. arenarium*, and *L. madreporoides*) from further analysis, in order to avoid the appearance of extensive SNPs and indels.

### 3.5. IR Expansion and Contraction

**Figure 4:**
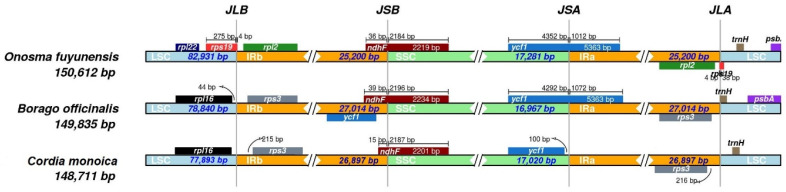
Comparison of the borders of LSC, SSC, and IR regions between *C. monoica*, *B. officinalis*, and *O. fuyunensis*. Genes are represented by colored boxes while arrows show the coordinate positions of each gene near the junctions. Abbreviations denote the junction site of the plastid genome JLA (IRa/LSC), JLB (IRb/LSC), JSA (SSC/IRa), and JSB (IRb/SSC).

Although the IR region of the chloroplast genome is the most conserved region, it is the border region contractions and expansions that are responsible for the variability in chloroplast genome length during evolution. The junction sites between each region are denoted as JLB (IRb/LSC), JSA (SSC/IRa), JSB (IRb/SSC), and JLA (IRa/LSC). In the current study, a comprehensive assessment of the four junctions (JLA, JLB, JSA, JSB) between *C. monoica, B. officinalis*, and *O. fuyunensis* was performed (Figure 4). The size variations in the plastomes causes dynamic changes in IR boundaries. The JLB boundary is similar in *C. monoica* and *B. officinalis* in terms of position and gene synteny and is located between *rpl*16 and *rps*3. This is in contrast to *O. fuyunensis,* located after the *rpl*16, with the *rps*3 junction toward *rpl*22, *rps*19, and *rpl*2. The JSB boundary is located within the *ndh*F gene in all the three species. The ycf1 gene is crossed by the JSA boundary in *O. fuyunensis* and *B. officinalis* but not in *C. monoica*. The JLA boundary is located between *rps*3 and trnH in *B. oficianlis* and *C. monoica*, in contrast to *O. fuyunensis,* where the boundary is located between *rpl*2 and *rps*19.

### 3.6. SNPs, Indels, and Selective Pressure Analysis

Using the *O. fuyunensis* cp genome as the reference sequence, the single nucleotide polymorphism (SNP) and indels (insertion and deletion) loci of the *C. monoica*, *B. officinalis*, and *O. fuyunensis* were assessed across the protein-coding genes. The results reveal a total of 5580 variations, including 5398 SNPs and 113 indels (55 deletions and 58 insertions). Of these indels, 30 (26.5%) are single-base indels, and the indel size ranges from 1 bp to 21 bp. The most abundant indel sites are detected in the IR region, followed by the SSC and LSC regions, while the highest numbers of indel are recorded in ycf1, ycf2, and rpoC2. All SNPs are classified into two types: synonymous (dS) and nonsynonymous (dN). There are 3050 synonymous SNPs and 2348 nonsynonymous SNPs in the protein-coding genes. The LSC region contains the majority of the SNPs (48%), followed by the SSC region (29%), and the IR region (15%). The most substitutions are found in the rpoC2 gene, followed by the ycf1 and ycf2 genes.

To detect the selective pressure on the PCGs of *C. monoica*, *B. officinalis*, and *O. fuyunensis* cp genomes, the rates of synonymous (dS) and nonsynonymous (dN) substitutions, and the dN/dS ratio were calculated. The dS values ranges from 0 (*psb*L) to 376 (rpoc2), with a total average value of 39.39, while the dN values ranges from 0 (*pbf*1, *pet*N, *psa*J, *psb*F, *psb*M, *psb*I) to 519 (*ycf*1) with a total average value of 31.32. Most dN/dS ratios are less than 1, possibly indicating that most cp genes are under purifying selection. Twelve cp genes, including *rps*15, *ccs*A, *ndh*F, *psb*H, *rps*7, *rpo*A, *rps*16, *rpl*23, *psb*K, *mat*K, *ycf*1, and *ycf*2 are detected with dN/dS values  >  1, indicating that these genes undergo a positive selection and only four genes (*psa*l, *psb*T, *rpl*33, and *rpl*36) have dN/dS values = 1.

### 3.7. Phylogenetic Analysis

**Figure 5:**
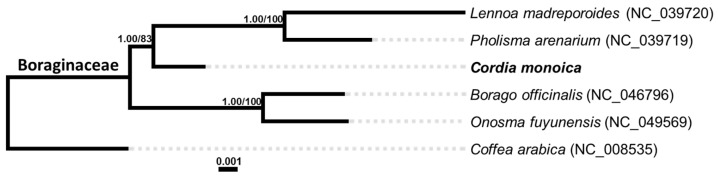
Phylogenetic trees of *C. monoica* and other species belong to the Boraginaceae family using maximum likelihood based on the LSC region, SSC region, and IR region together. The bootstrap values of ML and MP are written on each node.

To clarify the relationship between five Boraginaceae species, phylogenetic trees were constructed based on the sequences of the LSC region, the SSC region, and the IR region together (Figure 5). The results of ML/MP analyses based on the three regions yielded identical topologies with generally high support values. In the phylogeny tree, the five Boraginaceae species can be divided into two well-supported clades. Interestingly, the *P. arenarium* is grouped with *L. madreporoides* in the same clade, both are heterotrophs and parasitic plants, and *C. monoica* is placed as a sister group in the ingroup, while *B. officinalis* and *O. fuyunensis* form the other clade.

## 4. Discussion

Chloroplast genomes have been used for taxonomic and evolutionary studies to evaluate evolutionary relationships and determine genome structure, especially among closely related species [36,37]. This study sequenced and assembled the first complete cp genome from *C. monoica*, which was sampled from the Faifa mountains in Saudi Arabia. For the comparative analysis, four additional Boraginaceae chloroplast genomes were combined from the GenBank database. This study contributes to the database’s ever-expanding resources and is valuable for further studies on molecular identification, genetic diversity, and phylogenetics related to Boraginaceae.

The *C. monoica* cp genome typically exists as a double-stranded circular molecule with two inverted repeats (IR) and one large single copy (LSC) [38,39]. Our assembly and annotation results show that the *C. monoica* cp genome length is 148,711 bp, which is in the range of other Boraginaceae species [40,41], displaying similar genome structures and gene arrangements. While the tRNA and rRNA gene compositions of the three Boraginaceae species are similar, some differences are observed in the number of PCGs. The cp genomes of *C. monoica* are found to encode 89 PCGs, whereas *O. fuyunensis* and *B. officinalis* possess 84, and 83 PCGs, respectively. In this case, the variation occurs due to the pseudogenization and location of *ycf*1 and *rpl*23 in the IR region. Angiosperm cp genomes evolve relatively fast, and gene losses and inversions occur during their evolution [42].

Comparing the *C. monoica* cp genome with four related species, the sizes of the two Boraginaceae chloroplast genomes (*P. arenarium* and *L. madreporoides*) are significantly shorter than those of most angiosperms. Most angiosperm chloroplast genomes are 120 to 160 kb in length [43], while the sizes of the chloroplast genomes of *P. arenarium* and *L. madreporoides* range from 81,198 to 83,657 bp. Compared with most angiosperms, the sizes of the four regions of *P. arenarium* and *L. madreporoides* change significantly, and the most conspicuous change occurs in LSC and SSC, reduced by about 40 and 10 kb in size, respectively. Thus, these two Boraginaceae species have smaller chloroplast genomes because of the expansion of IRs. Several chloroplast genomes have been reported, which are significantly smaller than most other plants. Usually, small chloroplast genomes are found in parasitic plants, such as *Epifagus virginiana* in Orobanchaceae of Lamiales [11], and *Cuscuta chinensis* in Convolvulaceae of Solanales [44].

At lower taxonomic levels, tandem repeats have been shown to be an important molecular marker for species discrimination and population genetics [45]. Additionally, they have been used in a wide range of studies, including estimating genetic variation, analyzing gene flow, and exploring animal and plant populations [46,47]. Previously reported findings agree with those of the present study. In chloroplast genomes, poly-A or poly-T repeats are combined with tandem guanine or cytosine repeats [48], resulting in AT-rich chloroplast genomes [49,50].

Using codons correctly plays an essential role in expressing genetic information [51], resulting in a correlation between gene expression level, GC content, amino acid conservation, and transcriptional selection [52]. The most frequent are codons encoding leucine, and the least frequent are codons encoding cysteine. This result was confirmed in different species, such as *Cinnamomum camphora* [53] and Ocotea species [54]. As found in most chloroplast genomes from land plants, the codon preference for A/U codons is stronger than that for G/C codons [55,56].

A dynamic expansion or contraction of the four IR boundaries frequently occurs during the evolution of cp genomes, which results in further changes in the cp genome size. Researchers previously discovered that chloroplast genome size can change as a result of gene deletions [57] and intergenic variation [58], as well as contraction or expansion of the IR regions [59]. Due to their contraction and expansion at the borders, IR regions explain size variation between cp genomes despite being the most conserved in cp genome sequences [60,61,62,63].

In spite of the highly conserved genome of the cp, SNPs are clustered in “hotspots” [64], resulting in highly variable loci. In addition, variable hotspots containing indels have also been reported [65]. It is likely that the hotspots in the cp genome produce several highly variable cp genome markers. In contrast to commonly used molecular markers, the cp genome has a conserved sequence length of 110 to 160 kb, allowing for greater variation between closely related species [66]. A significant amount of structural variation (SNPs and indels) is found across cp genomes. As a result, some mutation hotspot regions could be tested as DNA markers specific to Boraginaceae (i.e., the coding genes, *mat*K, *rbc*L, and *rpl*16; the non-coding regions, rps16 intron; and the intergenic spacers *rps*18-*rpl*22, *trn*M-*ycf*2, *rps*15-*ycf*1, *ycf*1, *trn*V-*rps*12, and *trn*M-*rpl*23). In this list, *mat*K and *rbc*L are known as standard DNA barcode sequences. The genetic variation within these regions might also be sufficient to resolve the phylogenetic relationship of Boraginaceae species.

It is important to analyze the adaptive evolution of genes to understand how the substitution rate impacts the alteration of gene structure and function. An estimation of the dN/dS ratio can give insight into the constraints on organisms imposed by natural selection [67,68]. A sequence divergence analysis of protein-coding genes was conducted in the present study, and twelve of them (*rps*15, *ccs*A, *ndh*F, *psb*H, *rps*7, *rpo*A, *rps*16, *rpl*23, *psb*K, *mat*K, *ycf*1, and *ycf*2) show a difference between dN and dS of >1, which is expected of genes under positive selection. Among these, *rpl* and *rps* encode ribosomal proteins that have more divergent sequences than proteins related to photosynthesis [69], the *psb*H gene is associated with photosystem II [70], the *mat*K gene is involved in the cutting/splicing of group II RNA transcriptional introns [71], the *rpo*A encoding proteins are involved in transcription [72] and the *ccs*A encoding proteins are involved in the cytochrome synthesis gene [73]. Furthermore, the *psb*K and *ndh*F genes show photosynthesis-linked roles, indicating their role in photosynthesis and carbon fixation [74,75]. The genes *ycf*1 and *ycf*2 are two of the largest genes encoding for a putative membrane protein [76,77]. All of these genes are essential for plants to adapt to their environments and survive [78].

In the past two decades, a number of studies using chloroplast DNA have greatly enhanced our understanding of evolutionary relationships among angiosperms using cp DNA sequences [79]. The present study uses ML and MP analyses of different datasets to construct a phylogenetic tree with similar topological structures. As a result of the phylogenetic analysis, it is possible to delimit species by paraphyletic clustering based on their genetic variation. However, the large deletions found among the studied accessions violate the molecular clock assumptions and impede the ability to infer the divergence time accurately [80]. However, a much larger number of sequences are necessary to obtain a more accurate relationship between the Boraginaceae.

## 5. Conclusions

The complete chloroplast of *C. monoica* species was sequenced, assembled, and compared. The chloroplast genomes of *C. monoica* are conserved in terms of structure and gene order. Tandem repeats are found in the noncoding regions that might be useful for studying population genetics within the family Boraginaceae. A number of high-variability hotspots are also detected in the protein-coding genes for Boraginaceae species, which provide candidates for genetic markers for species identification and phylogeny. Additionally, three closely related species were compared in terms of their IR expansion and contraction. Analysis of coding gene sequence divergence reveals that twelve genes are positively selected. As a result of the study, the data obtained are helpful for future research on Boraginaceae diversity, ecology, taxonomy, phylogenetic evolution, and conservation.

## Figures and Tables

**Table 1:** Summary of the newly assembled chloroplast genome of the *C. monoica*.

Feature	*C. monoica*	Feature	*C. monoica*
Total cp DNA size (bp)	148,711	Intergenic sequences (%)	39.0%
LSC size (bp)	77,893	Number of genes	133
SSC size (bp)	17,020	Number of different protein-coding genes	90
IR size (bp)	26,897	Number of different tRNA genes	37
Protein-coding regions (%)	52.98%	Number of different rRNA genes	8
rRNA and tRNA (%)	7.94%	Number of different duplicated genes	21
Introns size (% total)	13.58%	GC content	38.2%

**Table 2:** Repeated sequences in the *C. monoica* chloroplast genomes, including repeat class, repeat abundances, and percentage abundance.

Repeat Class	Repeat Abundances	Abundance (%)
Dinucleotide	110	8%
Trinucleotide	194	14%
Tetranucleotide	241	17%
Pentanucleotide	281	20%
Hexanucleotide	388	28%
7-nucleotide	77	6%
8-nucleotide	47	3%
9-nucleotide	30	2%
10-nucleotide	13	1%
Total	1387	100.00

**Table 3:** Summary of the cp lengths, variation in cp regions, and similarity percentage to *C. monoica* of five Boraginaceae species.

Species	Total Length	LSC bp	SSC bp	IR bp	Similarity%	Accession No.
*Cordia monoica*	148,711	77,893	17,020	26,897	100%	-
*Borago officinalis*	149,835	78,840	16,967	27,014	88%	NC_046796
*Onosma fuyunensis*	150,612	82,931	17,281	25,200	84.8%	NC_049569
*Pholisma arenarium*	81,198	30,262	6454	22,241	39.8%	NC_039719
*Lennoa madreporoides*	83,675	30,881	6830	22,982	40%	NC_039720

## Data Availability

The complete sequence of *C. monoica* was deposited into the NCBI GenBank, accession number OP224515.
